# The genome sequence of the sycamore periphyllus aphid, *Periphyllus acericola *(Walker, 1848)

**DOI:** 10.12688/wellcomeopenres.23341.1

**Published:** 2024-11-12

**Authors:** Liam M. Crowley, University of Oxford and Wytham Woods Genome Acquisition Lab, Darwin Tree of Life Barcoding collective, Wellcome Sanger Institute Tree of Life Management, Samples and Laboratory team, Wellcome Sanger Institute Scientific Operations: Sequencing Operations, Wellcome Sanger Institute Tree of Life Core Informatics team, Tree of Life Core Informatics collective, Darwin Tree of Life Consortium

**Affiliations:** 1 University of Oxford, Oxford, England, UK

**Keywords:** Periphyllus acericola, sycamore periphyllus aphid, genome sequence, chromosomal, Hemiptera

## Abstract

We present a genome assembly from an individual female *Periphyllus acericola* (the sycamore periphyllus aphid; Arthropoda; Insecta; Hemiptera; Aphididae). The genome sequence has a total length of 405.30 megabases. Most of the assembly is scaffolded into 9 chromosomal pseudomolecules, including the X sex chromosome. The mitochondrial genome has also been assembled and is 33.63 kilobases in length. Gene annotation of this assembly on Ensembl identified 21,463 protein-coding genes.

## Species taxonomy

Eukaryota; Opisthokonta; Metazoa; Eumetazoa; Bilateria; Protostomia; Ecdysozoa; Panarthropoda; Arthropoda; Mandibulata; Pancrustacea; Hexapoda; Insecta; Dicondylia; Pterygota; Neoptera; Paraneoptera; Hemiptera; Sternorrhyncha; Aphidomorpha; Aphidoidea; Aphididae; Chaitophorinae; *Periphyllus*; *Periphyllus acericola* (Walker, 1848) (NCBI:txid1425412).

## Background

*Periphyllus* species are medium to large, pear-shaped aphids that can be either winged or wingless. The genus comprises approximately 42 species, predominantly associated with maple and sycamore trees ( *Acer* spp.) in the family Sapindaceae ( InfluentialPoints, 2024). *Periphyllus acericola* (Walker, 1848), commonly known as the sycamore periphyllus aphid, primarily feeds on the undersides of leaves, petioles, and young shoots of the sycamore ( *Acer pseudoplatanus*). Its distribution is mainly confined to the United Kingdom and parts of Europe, with records indicating a more limited range than some congeners ( GBIF Secretariat, 2023).

The life cycle of *P. acericola* is complex. In spring, overwintering eggs hatch into wingless fundatrices, which reproduce parthenogenetically, leading to rapid population increases. Multiple generations of viviparous females occur throughout the growing season. As autumn approaches, sexual forms – males and oviparous females – are produced. After mating, females lay eggs on sycamore bark, which overwinter and hatch the following spring, continuing the cycle ( InfluentialPoints, 2024). This reproductive strategy enables the aphid to form large populations quickly, potentially causing leaf curling and impacting tree health ( NatureSpot, 2024).

Here, we present a chromosomal-level genome sequence for *Periphyllus acericola*, the first for this genus, based on a female specimen from Wytham Woods, Oxfordshire, UK.

## Genome sequence report

**Figure 1.**
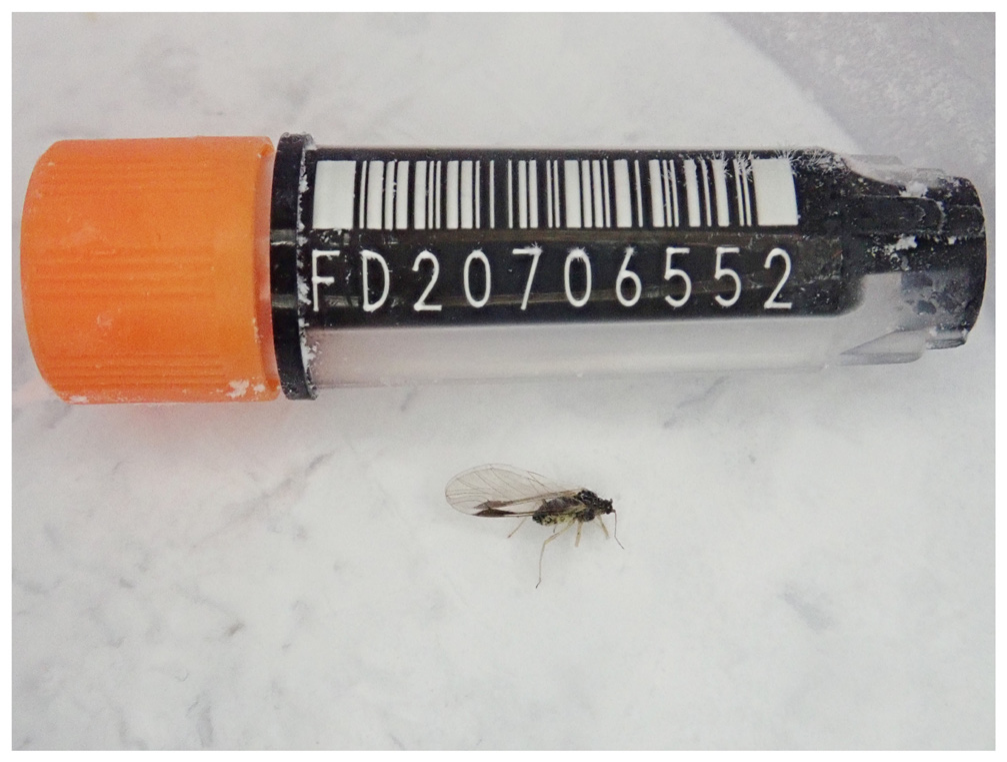
Photograph of the *Periphyllus acericola* (ihPerAcec1) specimen used for genome sequencing.

The genome of an adult female *Periphyllus acericola* ( Figure 1) was sequenced using Pacific Biosciences single-molecule HiFi long reads, generating a total of 19.40 Gb (gigabases) from 2.14 million reads, providing approximately 51-fold coverage. Primary assembly contigs were scaffolded with chromosome conformation Hi-C data, which produced 100.85 Gb from 667.86 million reads. Specimen and sequencing details are provided in Table 1.

**Table 1.** Specimen and sequencing data for *Periphyllus acericola*.

Project information			
**Study title**	*Periphyllus acericola* (sycamore periphyllus aphid)		
**Umbrella BioProject**	PRJEB60305		
**Species**	*Periphyllus acericola*		
**BioSample**	SAMEA10167089		
**NCBI taxonomy ID**	1425412		
Specimen information			
**Technology**	**ToLID**	**BioSample accession**	**Organism part**
**PacBio long read sequencing**	ihPerAcec1	SAMEA10201344	Whole organism
**Hi-C sequencing**	ihPerAcec2	SAMEA10201345	Whole organism
**RNA sequencing**	ihPerAcec3	SAMEA10201346	Whole organism
Sequencing information			
**Platform**	**Run accession**	**Read count**	**Base count (Gb)**
**Hi-C Illumina NovaSeq 6000**	ERR10968289	6.68e+08	100.85
**PacBio Sequel IIe**	ERR10962204	2.14e+06	19.4
**RNA Illumina NovaSeq 6000**	ERR12035181	5.89e+07	8.89

Manual assembly curation corrected 40 missing joins or mis-joins and three haplotypic duplications, reducing the scaffold number by 4.09%. The final assembly has a total length of 405.30 Mb in 468 sequence scaffolds with a scaffold N50 of 40.6 Mb ( Table 2).

**Table 2.** Genome assembly data for *Periphyllus acericola*, ihPerAcec1.1.

Genome assembly		
Assembly name	ihPerAcec1.1	
Assembly accession	GCA_949715065.1	
*Accession of alternate haplotype*	*GCA_949716515.1*	
Span (Mb)	405.30	
Number of contigs	809	
Number of scaffolds	468	
Longest scaffold (Mb)	70.63	
Assembly metrics *		*Benchmark*
Contig N50 length (Mb)	1.5	*≥ 1 Mb*
Scaffold N50 length (Mb)	40.6	*= chromosome N50*
Consensus quality (QV)	59.8	*≥ 40*
*k*-mer completeness	100.0%	*≥ 95%*
BUSCO **	C:99.0%[S:98.2%,D:0.9%], F:0.4%,M:0.6%,n:2,510	*S > 90%, D < 5%*
Percentage of assembly mapped to chromosomes	88.76%	*≥ 90%*
Sex chromosomes	X	*localised homologous pairs*
Organelles	Mitochondrial genome: 33.63 kb	*complete single alleles*
Genome annotation of assembly GCA_949715065.1 at Ensembl		
Number of protein-coding genes	21,463	
Number of gene transcripts	21,697	

* Assembly metric benchmarks are adapted from column VGP-2020 of “Table 1: Proposed standards and metrics for defining genome assembly quality” from Rhie *et al.* (2021). ** BUSCO scores based on the hemiptera_odb10 BUSCO set using version 5.3.2. C = complete [S = single copy, D = duplicated], F = fragmented, M = missing, n = number of orthologues in comparison. A full set of BUSCO scores is available at https://blobtoolkit.genomehubs.org/view/ihPerAcec1_1/dataset/ihPerAcec1_1/busco.

**Figure 2.**
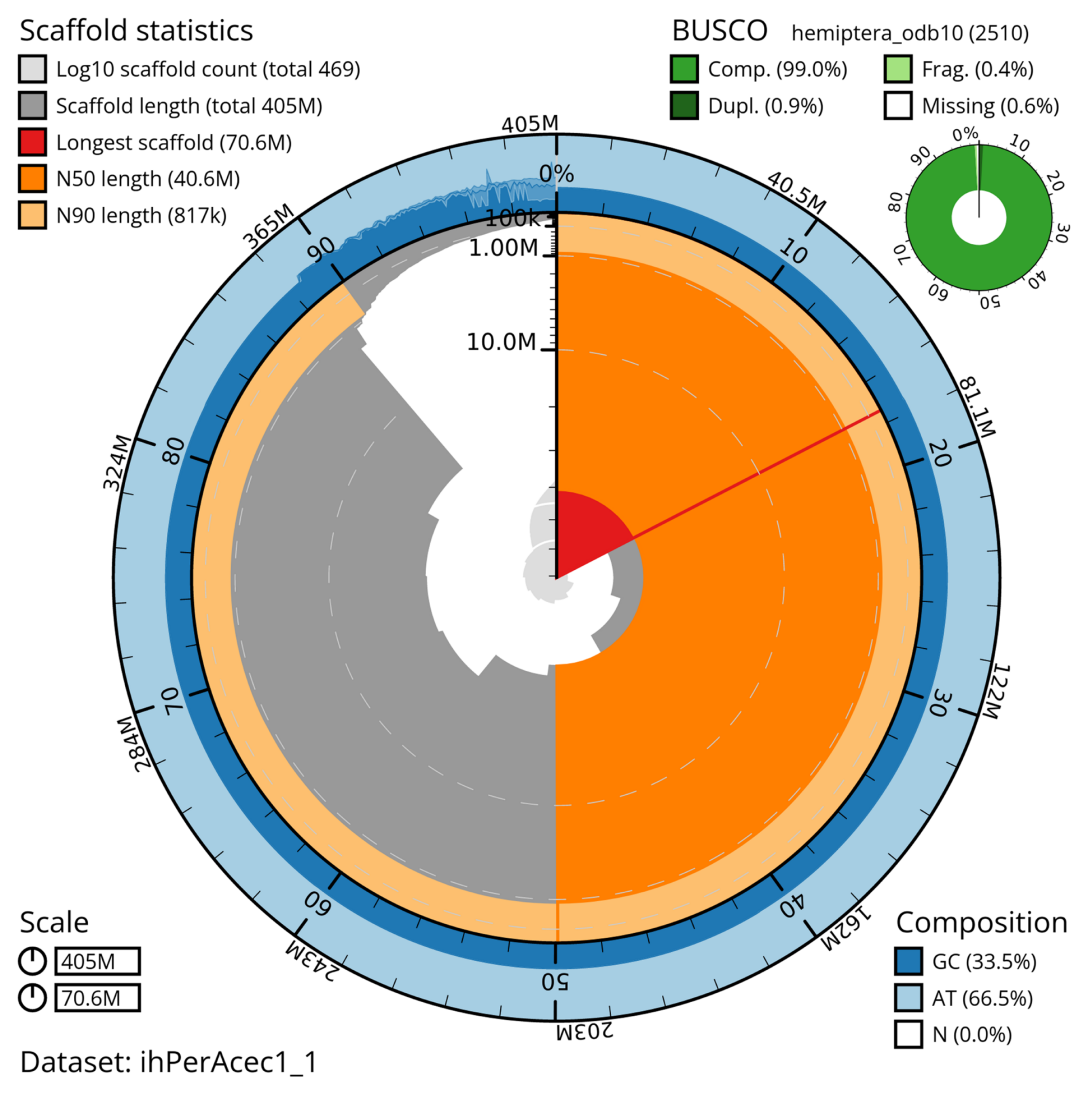
Genome assembly of *Periphyllus acericola*, ihPerAcec1.1: metrics. The BlobToolKit snail plot shows N50 metrics and BUSCO gene completeness. The main plot is divided into 1,000 size-ordered bins around the circumference with each bin representing 0.1% of the 405,379,013 bp assembly. The distribution of scaffold lengths is shown in dark grey with the plot radius scaled to the longest scaffold present in the assembly (70,630,091 bp, shown in red). Orange and pale-orange arcs show the N50 and N90 scaffold lengths (40,598,107 and 817,295 bp), respectively. The pale grey spiral shows the cumulative scaffold count on a log scale with white scale lines showing successive orders of magnitude. The blue and pale-blue area around the outside of the plot shows the distribution of GC, AT and N percentages in the same bins as the inner plot. A summary of complete, fragmented, duplicated and missing BUSCO genes in the hemiptera_odb10 set is shown in the top right. An interactive version of this figure is available at https://blobtoolkit.genomehubs.org/view/ihPerAcec1_1/dataset/ihPerAcec1_1/snail.

**Figure 3.**
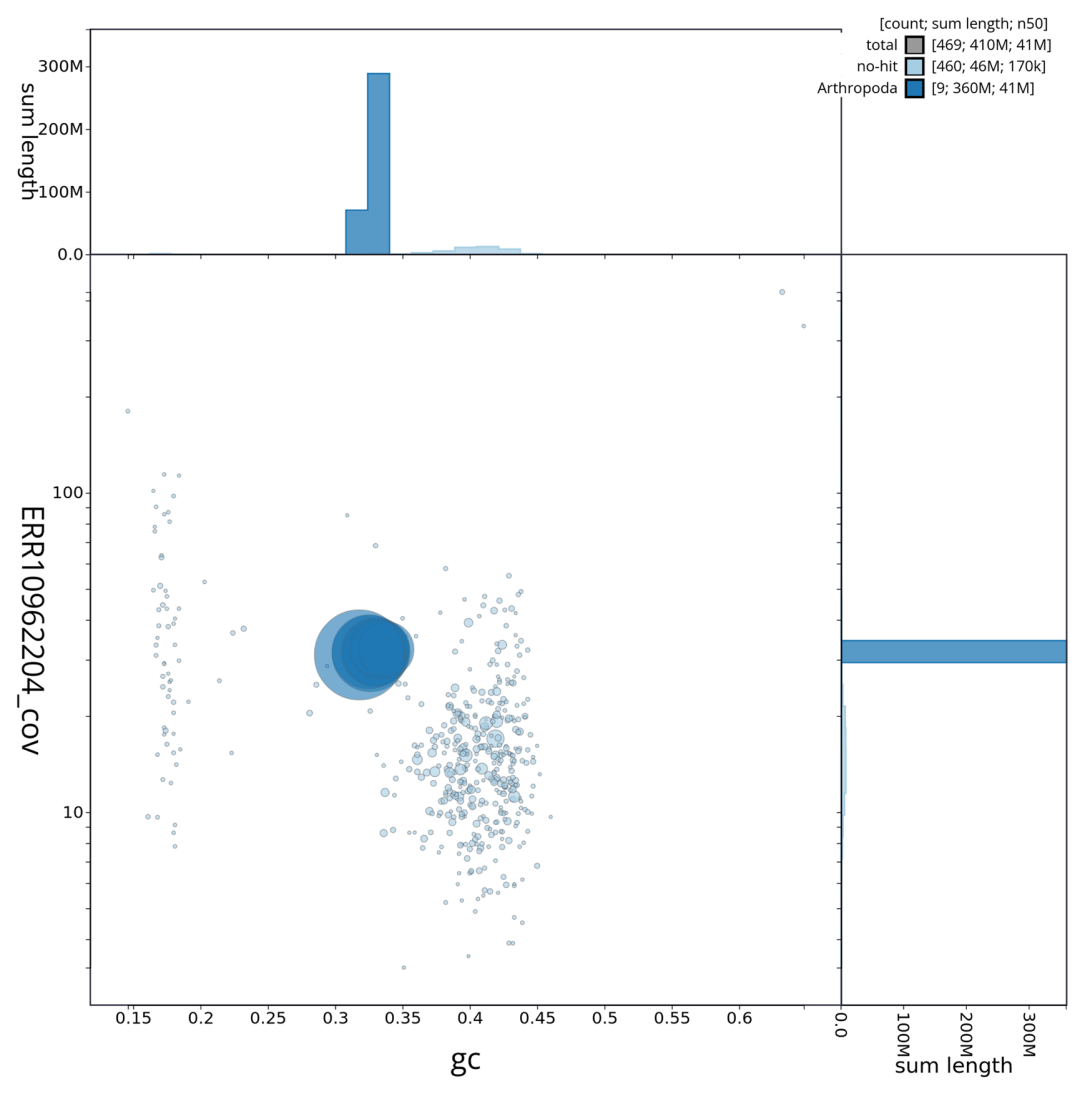
Genome assembly of *Periphyllus acericola*, ihPerAcec1.1: BlobToolKit GC-coverage plot. Blob plot showing sequence coverage (vertical axis) and GC content (horizontal axis). The circles represent scaffolds, with the size proportional to scaffold length and the colour representing phylum membership. The histograms along the axes display the total length of sequences distributed across different levels of coverage and GC content. An interactive version of this figure is available at https://blobtoolkit.genomehubs.org/view/ihPerAcec1_1/dataset/ihPerAcec1_1/blob.

**Figure 4.**
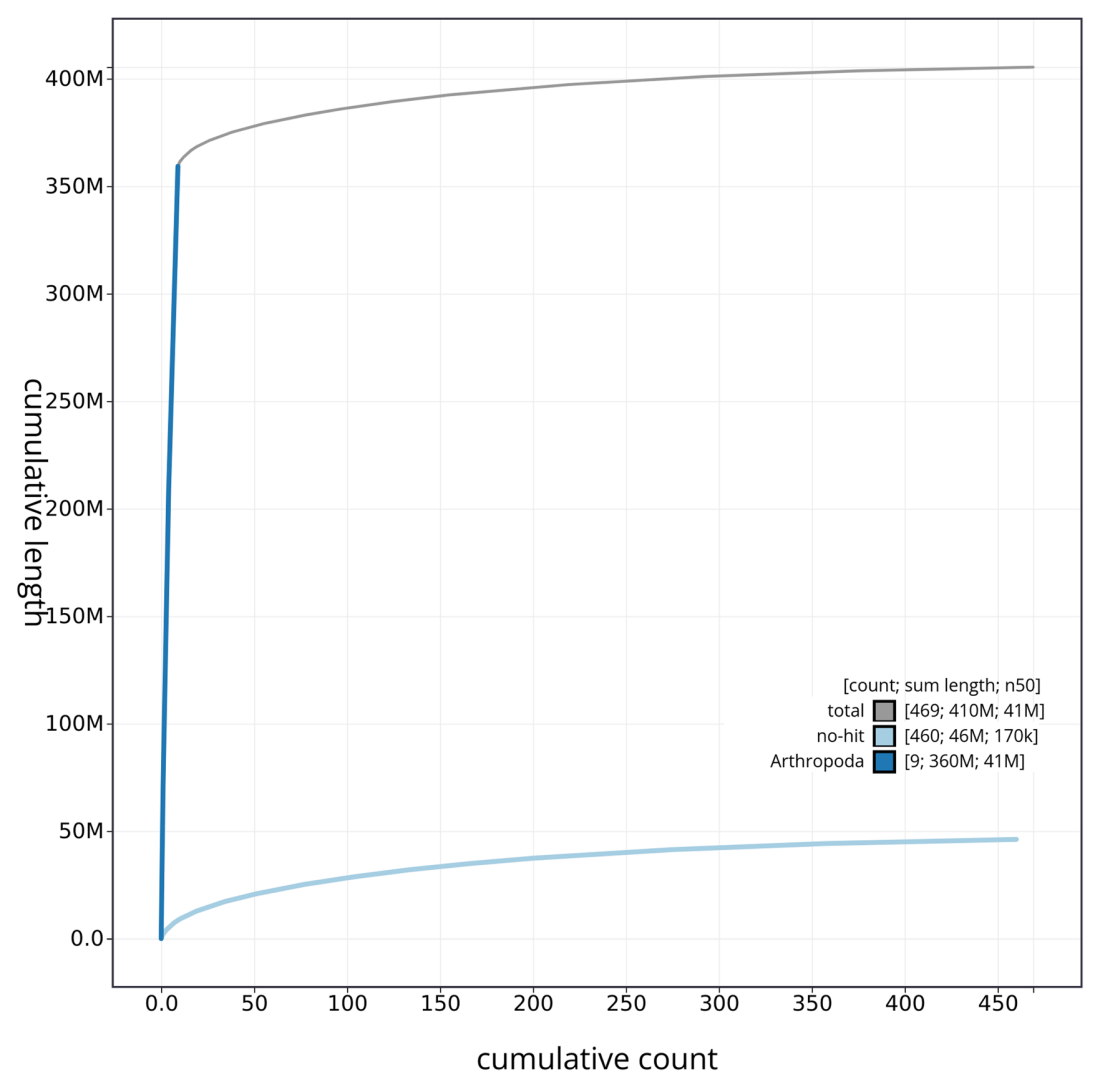
Genome assembly of *Periphyllus acericola* ihPerAcec1.1: BlobToolKit cumulative sequence plot. The grey line shows cumulative length for all sequences. Coloured lines show cumulative lengths of sequences assigned to each phylum using the buscogenes taxrule. An interactive version of this figure is available at https://blobtoolkit.genomehubs.org/view/ihPerAcec1_1/dataset/ihPerAcec1_1/cumulative.

**Figure 5.**
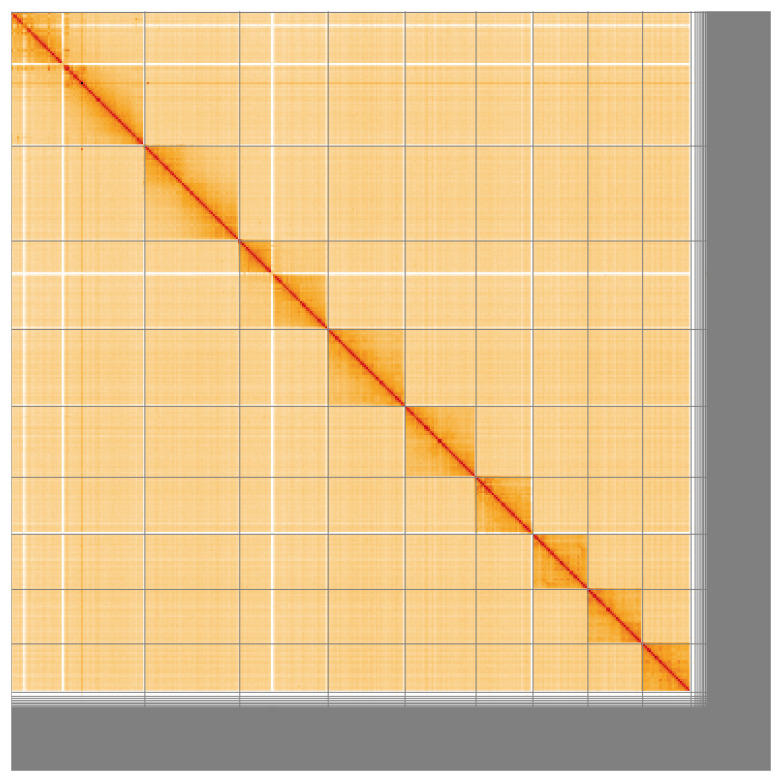
Genome assembly of *Periphyllus acericola*, ihPerAcec1.1: Hi-C contact map of the ihPerAcec1.1 assembly, visualised using HiGlass. Chromosomes are shown in order of size from left to right and top to bottom. An interactive version of this figure may be viewed at https://genome-note-higlass.tol.sanger.ac.uk/l/?d=S9L6SUtrRfSNc69WsZn8Kw.

The snail plot in Figure 2 provides a summary of the assembly statistics, while the distribution of assembly scaffolds on GC proportion and coverage is shown in Figure 3. The cumulative assembly plot in Figure 4 shows curves for subsets of scaffolds assigned to different phyla. Most (88.76%) of the assembly sequence was assigned to 9 chromosomal-level scaffolds, representing 8 autosomes and the X sex chromosome. Chromosome-scale scaffolds confirmed by the Hi-C data are named in order of size ( Figure 5; Table 3).

**Table 3.** Chromosomal pseudomolecules in the genome assembly of *Periphyllus acericola*, ihPerAcec1.

INSDC accession	Name	Length (Mb)	GC%
OX454265.1	1	50.07	32.5
OX454266.1	2	46.75	32.5
OX454267.1	3	40.6	33.0
OX454268.1	4	37.49	33.0
OX454269.1	5	30.12	33.5
OX454270.1	6	29.11	33.5
OX454271.1	7	28.78	33.0
OX454272.1	8	25.69	34.0
OX454264.1	X	70.63	32.0
OX454273.1	MT	0.03	14.5

During manual curation it was noted that the order and orientation of contigs on chromosome 2 from 0 to 1 Mb is uncertain. The X chromosome was identified based on synteny with the *Myzus persicae* clone O v2 genome assembly ( Mathers *et al.*, 2021). While not fully phased, the assembly deposited is of one haplotype. Contigs corresponding to the second haplotype have also been deposited. The mitochondrial genome was also assembled and can be found as a contig within the multifasta file of the genome submission.

The estimated Quality Value (QV) of the final assembly is 59.8 with *k*-mer completeness of 100.0%, and the assembly has a BUSCO v5.3.2 completeness of 99.0% (single = 98.2%, duplicated = 0.9%), using the hemiptera_odb10 reference set ( *n* = 2,510).

Metadata for specimens, BOLD barcode results, spectra estimates, sequencing runs, contaminants and pre-curation assembly statistics are given at https://links.tol.sanger.ac.uk/species/1425412.

## Genome annotation report

The *Periphyllus acericola* genome assembly (GCA_949715065.1) was annotated at the European Bioinformatics Institute (EBI) on Ensembl Rapid Release. The resulting annotation includes 21,697 transcribed mRNAs from 21,463 protein-coding genes ( Table 2; https://rapid.ensembl.org/Periphyllus_acericola_GCA_949715065.1/Info/Index). The average transcript length is 3,460.87. There are 1.01 coding transcripts per gene and 4.53 exons per transcript.

## Methods

### Sample acquisition and DNA barcoding

Adult specimens of *Periphyllus acericola* were netted in Wytham Woods, Oxfordshire (biological vice-county Berkshire), UK (latitude 51.76, longitude –1.33) on 2021-05-31. One specimen (specimen ID Ox001537, ToLID ihPerAcec1) was used for PacBio HiFi sequencing to generate the genome, another (specimen ID Ox001538, ToLID ihPerAcec2) was used for Hi-C scaffolding, and a third (specimen ID Ox001539, ToLID ihPerAcec3) was used for RNA sequencing. The specimens were collected and identified by Liam Crowley (University of Oxford) and were preserved on dry ice.

The initial identification was verified by an additional DNA barcoding process according to the framework developed by Twyford *et al*. (2024). A small sample was dissected from the specimens and stored in ethanol, while the remaining parts of the specimen were shipped on dry ice to the Wellcome Sanger Institute (WSI). The tissue was lysed, the COI marker region was amplified by PCR, and amplicons were sequenced and compared to the BOLD database, confirming the species identification ( Crowley *et al.*, 2023). Following whole genome sequence generation, the relevant DNA barcode region was also used alongside the initial barcoding data for sample tracking at the WSI ( Twyford *et al.*, 2024). The standard operating procedures for Darwin Tree of Life barcoding have been deposited on protocols.io ( Beasley *et al.*, 2023).

### Nucleic acid extraction

The workflow for high molecular weight (HMW) DNA extraction at the WSI Tree of Life Core Laboratory includes a sequence of core procedures: sample preparation and homogenisation, DNA extraction, fragmentation, and clean-up. Detailed protocols are available on protocols.io. In sample preparation, the ihPerAcec1 sample was weighed and dissected on dry ice ( Jay *et al.*, 2023). Tissue from the whole organism was homogenised using a PowerMasher II tissue disruptor ( Denton *et al.*, 2023).

HMW DNA was extracted using the Manual MagAttract v1 protocol ( Strickland *et al.*, 2023b). DNA was sheared into an average fragment size of 12–20 kb in a Megaruptor 3 system ( Todorovic *et al.*, 2023). Sheared DNA was purified by solid-phase reversible immobilisation, using of AMPure PB beads to eliminate shorter fragments and concentrate the DNA ( Strickland *et al.*, 2023a). The concentration of the sheared and purified DNA was assessed using a Nanodrop spectrophotometer and Qubit Fluorometer using the Qubit dsDNA High Sensitivity Assay kit. Fragment size distribution was evaluated by running the sample on the FemtoPulse system.

RNA was extracted from whole organism tissue of ihPerAcec3 in the Tree of Life Laboratory at the WSI using the RNA Extraction: Automated MagMax™ *mir*Vana protocol ( do Amaral *et al.*, 2023). The RNA concentration was assessed using a Nanodrop spectrophotometer and a Qubit Fluorometer using the Qubit RNA Broad-Range Assay kit. Analysis of the integrity of the RNA was done using the Agilent RNA 6000 Pico Kit and Eukaryotic Total RNA assay.

### Hi-C preparation

Tissue from the whole organism of ihPerAcec2 sample was processed at the WSI Scientific Operations core, using the Arima-HiC v2 kit. Frozen tissue (stored at –80 °C) was fixed, and the DNA crosslinked using a TC buffer with 22% formaldehyde. After crosslinking, the tissue was homogenised using the Diagnocine Power Masher-II and BioMasher-II tubes and pestles. Following the kit manufacturer's instructions, crosslinked DNA was digested using a restriction enzyme master mix. The 5’-overhangs were then filled in and labelled with biotinylated nucleotides and proximally ligated. An overnight incubation was carried out for enzymes to digest remaining proteins and for crosslinks to reverse. A clean up was performed with SPRIselect beads prior to library preparation.

### Library preparation and sequencing

Library preparation and sequencing were performed at the WSI Scientific Operations core. Pacific Biosciences HiFi circular consensus DNA sequencing libraries were prepared using the PacBio Express Template Preparation Kit v2.0 (Pacific Biosciences, California, USA) as per the manufacturer’s instructions. The kit includes the reagents required for removal of single-strand overhangs, DNA damage repair, end repair/A-tailing, adapter ligation, and nuclease treatment. Library preparation also included a library purification step using AMPure PB beads (Pacific Biosciences, California, USA) and size selection step to remove templates shorter than 3 kb using AMPure PB modified SPRI. DNA concentration was quantified using the Qubit Fluorometer v2.0 and Qubit HS Assay Kit and the final library fragment size analysis was carried out using the Agilent Femto Pulse Automated Pulsed Field CE Instrument and gDNA 165kb gDNA and 55kb BAC analysis kit. Samples were sequenced using the Sequel IIe system (Pacific Biosciences, California, USA). The concentration of the library loaded onto the Sequel IIe was between 40–135 pM. The SMRT link software, a PacBio web-based end-to-end workflow manager, was used to set-up and monitor the run, as well as perform primary and secondary analysis of the data upon completion.

For Hi-C library preparation, DNA was fragmented to a size of 400 to 600 bp using a Covaris E220 sonicator. The DNA was then enriched, barcoded, and amplified using the NEBNext Ultra II DNA Library Prep Kit following manufacturers’ instructions. The Hi-C sequencing was performed using paired-end sequencing with a read length of 150 bp on an Illumina NovaSeq 6000 instrument.

Poly(A) RNA-Seq libraries were constructed using the NEB Ultra II RNA Library Prep kit, following the manufacturer’s instructions. RNA sequencing was performed on the Illumina NovaSeq 6000 instrument.

### Genome assembly, curation and evaluation

***Assembly***

The original assembly of HiFi reads was performed using Hifiasm ( Cheng *et al.*, 2021) with the --primary option. Haplotypic duplications were identified and removed with purge_dups ( Guan *et al.*, 2020). Hi-C reads are further mapped with bwamem2 ( Vasimuddin *et al.*, 2019) to the primary contigs, which are further scaffolded using the provided Hi-C data ( Rao *et al.*, 2014) in YaHS ( Zhou *et al.*, 2023) using the --break option. Scaffolded assemblies are evaluated using Gfastats ( Formenti *et al.*, 2022), BUSCO ( Manni *et al.*, 2021) and MERQURY.FK ( Rhie *et al.*, 2020).

The mitochondrial genome was assembled using MitoHiFi ( Uliano-Silva *et al.*, 2023), which runs MitoFinder ( Allio *et al.*, 2020) and uses these annotations to select the final mitochondrial contig and to ensure the general quality of the sequence.

***Assembly curation***

The assembly was decontaminated using the Assembly Screen for Cobionts and Contaminants (ASCC) pipeline (article in preparation). Flat files and maps used in curation were generated in TreeVal ( Pointon *et al.*, 2023). Manual curation was primarily conducted using PretextView ( Harry, 2022), with additional insights provided by JBrowse2 ( Diesh *et al.*, 2023) and HiGlass ( Kerpedjiev *et al.*, 2018). Scaffolds were visually inspected and corrected as described by Howe *et al*. (2021). Any identified contamination, missed joins, and mis-joins were corrected, and duplicate sequences were tagged and removed. The entire process is documented at https://gitlab.com/wtsi-grit/rapid-curation (article in preparation).

***Evaluation of the final assembly***

A Hi-C map for the final assembly was produced using bwa-mem2 ( Vasimuddin *et al.*, 2019) in the Cooler file format ( Abdennur & Mirny, 2020). To assess the assembly metrics, the *k*-mer completeness and QV consensus quality values were calculated in Merqury ( Rhie *et al.*, 2020). This work was done using the “sanger-tol/readmapping” ( Surana *et al.*, 2023a) and “sanger-tol/genomenote” ( Surana *et al.*, 2023b) pipelines. The genome readmapping pipelines were developed using the nf-core tooling ( Ewels *et al.*, 2020), use MultiQC ( Ewels *et al.*, 2016), and make extensive use of the Conda package manager, the Bioconda initiative ( Grüning *et al.*, 2018), the Biocontainers infrastructure ( da Veiga Leprevost *et al.*, 2017), and the Docker ( Merkel, 2014) and Singularity ( Kurtzer *et al.*, 2017) containerisation solutions. The genome was also analysed within the BlobToolKit environment ( Challis *et al.*, 2020) and BUSCO scores ( Manni *et al.*, 2021) were calculated.

Table 4 contains a list of relevant software tool versions and sources.

**Table 4.** Software tools: versions and sources.

Software tool	Version	Source
BlobToolKit	4.2.1	https://github.com/blobtoolkit/blobtoolkit
BUSCO	5.3.2	https://gitlab.com/ezlab/busco
Hifiasm	0.16.1-r375	https://github.com/chhylp123/hifiasm
HiGlass	1.11.6	https://github.com/higlass/higlass
Merqury	MerquryFK	https://github.com/thegenemyers/MERQURY.FK
MitoHiFi	2	https://github.com/marcelauliano/MitoHiFi
PretextView	0.2	https://github.com/wtsi-hpag/PretextView
purge_dups	1.2.3	https://github.com/dfguan/purge_dups
sanger-tol/ genomenote	v1.0	https://github.com/sanger-tol/genomenote
sanger-tol/ readmapping	1.1.0	https://github.com/sanger-tol/readmapping/tree/1.1.0
YaHS	yahs-1.1.91eebc2	https://github.com/c-zhou/yahs

### Genome annotation

The BRAKER2 pipeline ( Brůna *et al.*, 2021) was used in the default protein mode to generate annotation for the *Periphyllus acericola* assembly (GCA_949715065.1) in Ensembl Rapid Release at the EBI.

### Wellcome Sanger Institute – Legal and Governance

The materials that have contributed to this genome note have been supplied by a Darwin Tree of Life Partner. The submission of materials by a Darwin Tree of Life Partner is subject to the **‘Darwin Tree of Life Project Sampling Code of Practice’**, which can be found in full on the Darwin Tree of Life website here. By agreeing with and signing up to the Sampling Code of Practice, the Darwin Tree of Life Partner agrees they will meet the legal and ethical requirements and standards set out within this document in respect of all samples acquired for, and supplied to, the Darwin Tree of Life Project. 

Further, the Wellcome Sanger Institute employs a process whereby due diligence is carried out proportionate to the nature of the materials themselves, and the circumstances under which they have been/are to be collected and provided for use. The purpose of this is to address and mitigate any potential legal and/or ethical implications of receipt and use of the materials as part of the research project, and to ensure that in doing so we align with best practice wherever possible. The overarching areas of consideration are:

•   Ethical review of provenance and sourcing of the material

•   Legality of collection, transfer and use (national and international)

Each transfer of samples is further undertaken according to a Research Collaboration Agreement or Material Transfer Agreement entered into by the Darwin Tree of Life Partner, Genome Research Limited (operating as the Wellcome Sanger Institute), and in some circumstances other Darwin Tree of Life collaborators.

## Data Availability

European Nucleotide Archive: *Periphyllus acericola* (sycamore periphyllus aphid). Accession number PRJEB60305; https://identifiers.org/ena.embl/PRJEB60305. The genome sequence is released openly for reuse. The *Periphyllus acericola* genome sequencing initiative is part of the Darwin Tree of Life (DToL) project. All raw sequence data and the assembly have been deposited in INSDC databases. Raw data and assembly accession identifiers are reported in Table 1 and Table 2.

## Author information

Members of the University of Oxford and Wytham Woods Genome Acquisition Lab are listed here: https://doi.org/10.5281/zenodo.12157525.

Members of the Darwin Tree of Life Barcoding collective are listed here: https://doi.org/10.5281/zenodo.12158331

Members of the Wellcome Sanger Institute Tree of Life Management, Samples and Laboratory team are listed here: https://doi.org/10.5281/zenodo.12162482.

Members of Wellcome Sanger Institute Scientific Operations: Sequencing Operations are listed here: https://doi.org/10.5281/zenodo.12165051.

Members of the Wellcome Sanger Institute Tree of Life Core Informatics team are listed here: https://doi.org/10.5281/zenodo.12160324.

Members of the Tree of Life Core Informatics collective are listed here: https://doi.org/10.5281/zenodo.12205391.

Members of the Darwin Tree of Life Consortium are listed here: https://doi.org/10.5281/zenodo.4783558.
