# Supplementary material for: The genome sequence of the sycamore periphyllus aphid, Periphyllus acericola (Walker, 1848)
Source: Wellcome Open Res. 2024 Nov 12;9:676. [Version 1] doi: 10.12688/wellcomeopenres.23341.1 (PMC11612552; doi:10.12688/wellcomeopenres.23341.1)
Supplement: Peer review history

# Reviewer report: Reviewer response for version 1

**DOI:** 10.21956/wellcomeopenres.25726.r111527

**Published:** 2024-12-02

**Authors:** Li Lim

**Affiliations:** 1 Universiti Sains Malaysia, George Town, Malaysia

This manuscript provides a detailed and well-structured presentation of the genome assembly for *Periphyllus acericola*. The study's rationale is clearly articulated, and the methodology is robust. The findings are significant, as this is the first genome assembly for this genus. However, the inclusion of a higher-resolution photograph of the aphid specimen would enhance the manuscript by better showcasing its morphological features. Overall, this is a commendable contribution to aphid genomics and insect biodiversity research.

Are sufficient details of methods and materials provided to allow replication by others?

Yes

Is the rationale for creating the dataset(s) clearly described?

Yes

Are the datasets clearly presented in a useable and accessible format?

Yes

Are the protocols appropriate and is the work technically sound?

Yes

Reviewer Expertise:

Molecular biology, entomology, plant pathology

I confirm that I have read this submission and believe that I have an appropriate level of expertise to confirm that it is of an acceptable scientific standard.

# Reviewer report: Reviewer response for version 1

**DOI:** 10.21956/wellcomeopenres.25726.r111530

**Published:** 2024-11-22

**Authors:** Qianquan Chen

**Affiliations:** 1 Guizhou Normal University, Guiyang, Guizhou, China

*Periphyllus acericola *(Walker, 1848) distributes in the United Kingdom and parts of Europe. It primarily feeds on the undersides of leaves, petioles, and young shoots of the sycamore. It exhibits clear phenotypic plasticity, i.e., individuals of *P. acericola* can be either winged or wingless. Furtherly, in spring, eggs hatch into wingless fundatrices, which reproduce parthenogenetically. In autumn, sexual forms (males and oviparous females) are produced to lay eggs. The genome sequence can contribute to unveil the molecular mechanism of phenotypic plasticity of *P. acericola*. I advise authors to use a new photo which can clearly show the morphological characteristics of the *P. acericola*.

Are sufficient details of methods and materials provided to allow replication by others?

Yes

Is the rationale for creating the dataset(s) clearly described?

Yes

Are the datasets clearly presented in a useable and accessible format?

Yes

Are the protocols appropriate and is the work technically sound?

Yes

Reviewer Expertise:

Eco-genomics

I confirm that I have read this submission and believe that I have an appropriate level of expertise to confirm that it is of an acceptable scientific standard.
